# Physicians' continuing medical education activities and satisfaction with their ability to stay current in medical information and practice: A cross‐sectional study

**DOI:** 10.1002/hsr2.1110

**Published:** 2023-02-10

**Authors:** Amy Jayas, Dorothy A. Andriole, Douglas Grbic, Xiaochu Hu, Michael Dill, Lisa D. Howley

**Affiliations:** ^1^ Academic Affairs Association of American Medical Colleges Washington District of Columbia USA

**Keywords:** continuing medical education, continuing professional development, demographics, satisfaction, specialty

## Abstract

**Background and Aims:**

Little is known about physicians' approaches to continuing medical education (CME) for continuing professional development despite the rapid evolution of CME offerings. We sought to identify the extent to which demographic, career, and experiential CME‐activity variables were independently associated with physicians' satisfaction with their ability to stay current on medical information and practice.

**Methods:**

Using the 2019 Association of American Medical Colleges' National Sample Survey of Physicians data, we ran multivariable logistic regression models examining demographic, career, and experiential (participation in 11 CME activities in the past year) variables for their associations with physicians' satisfaction (satisfied vs. not satisfied/neutral) with their ability to stay current.

**Results:**

Of 5926 respondents, 90% (5341/5926) were satisfied with their ability to stay current. Significant (each two‐sided *p* < 0.05) predictors of respondents who were satisfied included (among others) a surgery specialty (vs. primary care; adjusted odds ratio [AOR] = 1.41, 95% confidence interval [CI] = 1.06–1.88), an academic affiliation (vs. none; AOR = 1.35, 95% CI = 1.10–1.66), and participation (vs. no participation) in each of professional meetings (AOR = 1.31, 95% CI = 1.07–1.60) and journal‐based CME (AOR = 1.29, 95% CI = 1.07–1.56). Respondents who self‐identified as a race/ethnicity underrepresented in medicine (vs. white; AOR = 0.68, 95% CI = 0.48–0.97) and were between ages 40 and 49 years (vs. 50–59; AOR = 0.74; 95% CI = 0.58–0.94) were less likely to be satisfied. Gender, board certification status, and medical degree type did not independently predict satisfaction (each *p* > 0.05).

**Conclusion:**

We observed independent associations between physicians' satisfaction with their ability to stay current in medical information and practice and each specialty, academic affiliation, race/ethnicity, age, and CME activity type (for 2 of 11 examined). Findings may be relevant to organizations and institutions designing and implementing CME activities in the current context of COVID‐19 pandemic‐related in‐person activity limitations and can inform targeted interventions addressing differences in the satisfaction we observed to better support physicians' CME.

## 1 INTRODUCTION

As the amount of medical knowledge grows exponentially, staying current in numerous areas (e.g., scientific knowledge, technological advances, clinical practice innovations, and new delivery models) is critical for practicing physicians regardless of specialty or academic affiliation. Physicians, therefore, engage in continuing medical education (CME), a form of continuous professional development (CPD) defined as “educational activities which serve to maintain, develop, or increase the knowledge, skills, and professional performance and relationships that a physician uses to provide services for patients, the public, or the profession.”^1^ In the United States, most states require physicians to obtain CME credits to maintain state medical licenses, and most hospitals require a specified number of credits to maintain privileges to provide health care in their settings.

CME is delivered through a variety of modalities and approaches, frequently evolving due to many factors,^2^, ^3^ and further catalyzed by the COVID‐19 pandemic. The number of accredited CME activities offered continues to grow with approximately 179,000 activities reported in 2018 increasing to nearly 189,000 activities in 2019.^4^ Although there is rapid evolution and growth in CME offerings, our understanding of physicians' CME approaches (i.e., participation in CME activity types) and perspectives (i.e., satisfaction with the ability to stay current in medical information and practice), including the extent to which such perspectives may vary in association with physician demographic characteristics and professional attributes, is limited,^5^, ^6^ as are data on physician preferences in CME offerings in the United States.^6^, ^7^ The relationship between physicians' satisfaction with their ability to stay current on medical information and practice and the CME activities in which they choose to participate has not been explored in recent national studies,^5^, ^6^ which would contribute to the ongoing research that should collectively advance this field. A greater understanding of these adult learners' approaches to CME and their reported satisfaction with their ability to stay current on medical information and practice can aid in designing, delivering, and tailoring educational activities that support the learning preferences of diverse physicians across their professional careers.

In this study, we explored physician characteristics (demographics and professional) and CME approaches based on their participation in different CME activities, as reported in the 2019 Association of American Medical Colleges (AAMC) National Sample Survey of Physicians (NSSP), designed to capture national data on a collection of topics related to physician practice patterns and perspectives. We sought to determine nationally the extent to which these characteristics and CME approaches were independently associated with physicians' satisfaction with their ability to stay current on medical information and practice.

## 2 MATERIALS AND METHODS

This study examined data from the AAMC NSSP, a nationally representative survey of 6000 practicing physicians in the United States. collected between February and March 2019.^8^ The NSSP was conducted online for the AAMC by an external firm that recruited active physicians from their own and their partners' proprietary panels of 86,951 active physicians. Participants gave informed consent and received a cash incentive for survey completion.

To ensure the representation of the sample, AAMC used the American Medical Association's comprehensive database of physicians in the United States as a reference to set minimum sample sizes required by specialty, gender, and age based on power calculations. The survey was closed once the desired sample of 6000 participants was reached.^8^ The sampling error for the survey is ±1.3% at a 95% confidence level using a point estimate of 50%. Moreover, NSSP data were weighted based on the American Medical Association Physician database to represent all practicing physicians in the United States regarding specialty, gender, and age. This study was approved by the American Institute for Research's Institutional Review Board and deemed exempt from further review due to no human subject involvement and use of deidentified data, as defined in 45 CFR 46.

Physician respondents were asked a broad range of questions pertaining to (among other topics)^8^ their personal demographic characteristics, professional attributes, and CME. Our study sample consisted of all physicians with data for the set of demographic characteristics, professional attributes, and CME‐participation items of interest as described below. We examined these variables for their associations with our outcome of interest, physicians' satisfaction with their ability to stay current on medical information and practice. Demographic variables in our study included gender, age, and race/ethnicity. Although respondents in the survey were able to select nonbinary gender responses, due to the limited number responding in this way (<1%), the current study used a binary (male, female) gender variable for analyses, defining “male” as respondents who self‐identified as males or trans‐males and “female” as respondents who self‐identified as females or trans‐females. Physician age was categorized into four groups based on age in years: “less than 40,” “40–49,” “50–59,” and “60 and over.” In the context of widening recognition of racial/ethnic disparities in physician workforce training outcomes and the call to stratify outcomes‐reporting in academic medicine by race/ethnicity “to identify where disparities exist and to guide efforts to close gaps,”^9^ we examined race/ethnicity differences and categorized race/ethnicity into four groups based on “racial and ethnic populations that are underrepresented in the medical profession relative to their numbers in the general population,” as defined by the AAMC^10^: “underrepresented in medicine” (“URM,” including respondents who identified as Hispanic, Latino, or of Spanish origin; American Indian or Alaskan Native; Black or African American; or Native Hawaiian or other Pacific Islander alone or in combination with any other race/ethnicity), “Asian” (alone), “White” (alone), and “other” (Other alone; Asian and White; Asian and Other; White and Other; or Asian, White, and Other in combination).

Professional attributes in our study included medical degree type, specialty (defined as the specialty in which one spends the most time), board certification status in a given specialty, and current academic affiliation with an Academic Health Center or teaching hospital. A four‐category variable was created for medical degree type, including physicians who graduated with a “US/Canada [CA] medical degree,” “US osteopathic degree,” “medical degree from a non‐US/CA medical school,” and, to minimize loss of respondents in analyses, a “missing” category for respondents with missing/unknown medical degree type data. A four‐category variable was also created for physician specialty, including “primary care” (e.g., family medicine, general internal medicine, general pediatrics, and geriatric medicine), “medicine” (e.g., allergy and immunology, cardiology, critical care, dermatology, endocrinology, gastroenterology, hematology and oncology, infectious diseases, neonatal and perinatal medicine, nephrology, pulmonology, and rheumatology), “surgery” (e.g., general surgery, colorectal surgery, neurological surgery, obstetrics and gynecology, ophthalmology, orthopedic surgery, otolaryngology, plastic surgery, thoracic surgery, urology, vascular surgery, and other surgical specialties), and “other” (e.g., anesthesiology, emergency medicine, neurology, pathology, physical medicine and rehabilitation, psychiatry, radiology, and all other specialties).

The CME participation variable included in our study specifies the CME activities that physicians completed in the past year (from a drop‐down menu of 11 choices) to obtain CME credits. Given that multiple activities could be selected, we created a measure identifying the total number of different activities in which physicians participated in the past year or the “extent of CME activities participated”; this measure ranged from 0 to 10 CME activities participated. To obtain a sense of respondents' perspectives on their CME, our outcome of interest was based on dis/agreement (1–5 Likert scale response options [1 = strongly disagree to 5 = strongly agree]) with the statement, “overall, I am satisfied with my ability to stay current on medical information and practice.” Physician satisfaction with the ability to stay current on medical information and practice (referred to hereafter as “satisfaction with the ability to stay current”) was dichotomized to identify factors associated with physicians who were satisfied (i.e., those who “somewhat agreed” or “strongly agreed”) versus not satisfied/neutral (i.e., those who “somewhat disagreed,” “strongly disagreed,” or “neither agreed nor disagreed”) with their ability to stay current. We also employed an ordinal logit model to the data, and given the findings were essentially the same, we selected the binary logit model to use for this study.

We used *χ* ^2^ tests to assess the significance (two‐sided *p* < 0.05) of the relationship between physician satisfaction with the ability to stay current and each of the demographic characteristics, professional attributes, and CME‐participation items. We also assessed the association between satisfaction with the ability to stay current and the extent of participation in different CME‐activity types using the Pearson correlation coefficient and *p* < 0.05 as the threshold for statistical significance. We assessed two multivariable logistic regression models for independent associations between (1) participation in each CME activity and satisfaction with the ability to stay current and (2) the extent of CME‐activity participation and satisfaction with the ability to stay current. Model 1 includes physician demographic characteristics (gender, age category, and race/ethnicity category), professional attributes (specialty category, medical degree type, board certification status, and academic affiliation status), and participation in each of the 11 CME activities in the past year. Model 2 includes physician demographic characteristics (gender, age category, and race/ethnicity category), professional attributes (specialty category, medical degree type, board certification status, and academic affiliation status), and the variable created to measure the extent of CME activities participated in the past year. Model fit was assessed using the Archer–Lemeshow goodness‐of‐fit test ^11^ where poor fit is indicated by a *p* value of less than 0.05. Missing data were excluded from regression model analyses. All analyses were exploratory and were conducted using Stata version 15 (StataCorp).

## 3 RESULTS

Our final sample included 5926 (98.8% of all 6000 surveyed) physicians with data for all study variables (missing <1% for gender, academic affiliation, board certification, and satisfaction). Findings for the association between each of the demographic characteristics, professional attributes, and CME activities and our outcome are shown in Table 1. As shown, physicians were generally satisfied with their ability to stay current (90%; 5341/5926). Physicians who were satisfied with their ability to stay current were disproportionately male (91%, 3502/5341; *p* = 0.006), age ≥60 years (92%, 1755/5341; *p* = 0.001), self‐identified as white (91%, 3592/5341; *p* = 0.01), practiced in a surgery specialty (93%, 1036/5341; *p* = 0.001), and had an academic affiliation (92%, 2453/5341; *p* < 0.001). Medical degree type and board certification did not vary by satisfaction (Table 1; each *p* > 0.05).

**Table 1:** Physician demographic characteristics, professional attributes, and CME activities by their satisfaction with ability to stay current on medical information and practice.

	Total sample (*N* = 5926),^a^ *n* (%)^b^	Physicians satisfied with ability to stay current (*n* = 5341), *n* (%)^c^	Physicians not satisfied/neutral with ability to stay current (*n* = 585), *n* (%)^c^	*p* Value
*Demographic characteristics*				
Gender				
Female	2077 (35)	1838 (89)	238 (11)	0.006
Male	3849 (65)	3502 (91)	347 (9)	
Age (years) category				
<40	958 (16)	867 (91)	91 (10)	0.001
40–49	1553 (26)	1356 (87)	197 (13)	
50–59	1502 (25)	1363 (91)	139 (9)	
≥60	1913 (32)	1755 (92)	159 (8)	
Race/ethnicity category^d^				
White (alone)	3955 (67)	3592 (91)	363 (9)	0.01
URM (alone or in combination)	419 (7)	361 (86)	57 (14)	
Asian (alone)	1330 (22)	1196 (90)	134 (10)	
Other	222 (4)	191 (86)	31 (14)	
*Professional attributes*				
Specialty category				
Primary care	2066 (35)	1819 (88)	247 (12)	0.001
Medicine	993 (17)	891 (90)	101 (10)	
Surgery	1120 (19)	1036 (93)	84 (7)	
Other^e^	1748 (29)	1594 (91)	154 (9)	
Medical degree type				
US/CA medical degree	3798 (64)	3430 (90)	367 (10)	0.54
US osteopathic degree	506 (9)	448 (89)	57 (11)	
Medical degree from non‐US/CA medical school	1570 (26)	1412 (90)	157 (10)	
Missing	53 (<1)	50 (94)	3 (<1)	
Board certification				
Yes	5424 (92)	4897 (90)	527 (10)	0.24
No	502 (8)	444 (88)	59 (12)	
Academic affiliation				
Yes	2665 (45)	2453 (92)	213 (8)	<0.001
No	3261 (55)	2888 (89)	373 (11)	
*CME activities* f				
Professional meetings				
Participated	3735 (63)	3414 (91)	322 (9)	<0.001
Did not participate	2191 (37)	1927 (88)	264 (12)	
Journal‐based CME				
Participated	3392 (57)	3087 (91)	304 (9)	0.01
Did not participate	2534 (43)	2253 (89)	281 (11)	
Online specialty‐based courses				
Participated	3292 (56)	2964 (90)	328 (10)	0.83
Did not participate	2634 (44)	2376 (90)	258 (10)	
Local conferences				
Participated	2491 (42)	2262 (91)	229 (9)	0.15
Did not participate	3435 (58)	3078 (90)	357 (10)	
In‐person specialty‐based courses				
Participated	1975 (33)	1812 (92)	163 (8)	0.007
Did not participate	3951 (67)	3529 (89)	422 (11)	
Learning from teaching others				
Participated	1400 (24)	1278 (91)	122 (9)	0.11
Did not participate	4526 (76)	4063 (90)	464 (10)	
Performance improvement				
Participated	1380 (23)	1249 (91)	131 (9)	0.61
Did not participate	4546 (77)	4092 (90)	455 (10)	
Manuscript review				
Participated	689 (12)	649 (94)	40 (6)	0.001
Did not participate	5237 (88)	4691 (90)	546 (10)	
Committee learning				
Participated	645 (11)	593 (92)	52 (8)	0.11
Did not participate	5281 (89)	4747 (90)	533 (10)	
Test‐item writing				
Participated	577 (10)	527 (91)	51 (9)	0.40
Did not participate	5349 (90)	4814 (90)	535 (10)	
Other CME activity				
Participated	299 (5)	258 (86)	41 (14)	0.03
Did not participate	5627 (95)	5083 (90)	544 (10)	

*Note*: Associations between each of physician demographic characteristics, professional attributes, and CME activities with physicians' satisfaction with ability to stay current on medical information and practice. Each association was tested using χ^2^ test of association and considered statistically significant when the *p* value was less than 0.05.

Abbreviations: CA, Canada; CME, continuing medical education; URM, underrepresented in medicine; US, United States.

^a^ An analytical weight was applied to all analyses to ensure representativeness of all practicing physicians in the United States for specialty, gender, and age; in some cases, numbers were rounded to the nearest whole number and may not equal the total number per category. Unweighted *N* = 5930.

^b^ Column percentages sum up to 100% for each category; percentages may not equal 100% due to rounding. For each CME activity, column percentages sum up to 100% as respondents were able to select more than one activity.

^c^ Row percentages sum up to 100%; percentages may not equal 100% due to rounding.

^d^ “URM” includes Hispanic, Latino, or of Spanish origin; American Indian or Alaskan Native; Black or African American; or Native Hawaiian or other Pacific Islander alone or in combination with any other race/ethnicity. “Other” includes Other alone; Asian and White; Asian and Other; White and Other; or Asian, White, and Other in combination.

^e^ Top three specialties within “Other” include Psychiatry (14%, 252/1748), Anesthesiology (14%, 244/1748), and Emergency Medicine (13%, 219/1748).

^f^ Respondents selected as many CME activities participated in the past year (from a drop‐down menu of 11 choices) to obtain CME credits.

Participation in CME activities ranged from 5% (299/5,926) for “other” to 63% (3735/5926) for “professional meetings.” Satisfaction with ability to stay current varied by each of five CME activities: “professional meetings,” “journal‐based CME,” “in‐person specialty‐based courses,” “manuscript review,” and “other”; except for “other,” participants in each of these CME activities were overrepresented among those who were satisfied with their ability to stay current (Table 1; each *p* < 0.05). The remaining CME activities (“online specialty‐based courses,” “local conferences,” “learning from teaching others,” “performance improvement,” “committee learning,” and “test‐item writing”) did not vary by satisfaction (each *p* > 0.05). Not shown in Table 1, extent of participation in different CME‐activity types (mean = 3.35, SD = 1.73) correlated with satisfaction with ability to stay current (Pearson correlation = 0.081, *p* < 0.001).

Regression findings (Table 2) for demographic characteristics and professional attributes were similar in both models. Focusing on Model 1, physicians of ages 40–49 years (vs. 50–59; adjusted odds ratio [AOR], 0.74; 95% confidence interval [CI], 0.58–0.94) and self‐identified as a race/ethnicity underrepresented in medicine (vs. white; AOR, 0.68; 95% CI, 0.48–0.97) were less likely to be satisfied with their ability to stay current. Physicians in “surgery” (vs. “primary care” specialty category; AOR, 1.41; 95% CI, 1.06–1.88), in “other” specialties (vs. “primary care” specialty category; AOR, 1.30; 95% CI, 1.01–1.67), and with an academic affiliation (vs. none; AOR, 1.35; 95% CI, 1.10–1.66) were more likely to be satisfied with their ability to stay current. In Model 2, physicians self‐identified as “other” race/ethnicity (vs. white; AOR, 0.65; 95% CI, 0.43–0.98) were less likely to be satisfied. In either model, satisfaction with ability to stay current was not independently associated with each male, ages <40 and ≥60 years, Asian race/ethnicity, “medicine” specialty category, medical degree type, and board certification.

**Table 2:** Logistic regression results^a^ of physicians satisfied versus not satisfied/neutral with ability to stay current on medical information and practice.

	Model 1^b^	*p* Value^c^	Model 2^d^	*p* Value^c^
	**AOR (95% CI)**		**AOR (95% CI)**	
*Demographic characteristics*				
Gender				
Female	1 [Reference]	–	1 [Reference]	–
Male	1.19 (0.98–1.45)	0.07	1.21 (1.00–1.47)	0.06
Age (years) category				
<40	1.01 (0.74–1.38)	0.93	1.00 (0.73–1.36)	>0.99
40–49	0.74 (0.58–0.94)	0.01	0.74 (0.58–0.94)	0.02
50–59	1 [Reference]	–	1 [Reference]	–
≥60	1.10 (0.85–1.43)	0.48	1.11 (0.85–1.44)	0.44
Race/ethnicity category^e^				
White (alone)	1 [Reference]	–	1 [Reference]	–
URM (alone or in combination)	0.68 (0.48–0.97)	0.03	0.69 (0.49–0.98)	0.04
Asian (alone)	0.99 (0.77–1.28)	0.97	1.01 (0.79–1.30)	0.94
Other	0.65 (0.43–1.00)	0.05	0.65 (0.43–0.98)	0.04
*Professional attributes*				
Specialty category				
Primary care	1 [Reference]	–	1 [Reference]	–
Medicine	1.02 (0.79–1.32)	0.89	1.08 (0.84–1.39)	0.53
Surgery	1.41 (1.06–1.88)	0.02	1.50 (1.14–1.98)	0.004
Other^f^	1.30 (1.01–1.67)	0.05	1.30 (1.01–1.67)	0.04
Medical degree type				
US/CA medical degree	1 [Reference]	–	1 [Reference]	–
US osteopathic degree	0.89 (0.66–1.19)	0.43	0.91 (0.68–1.21)	0.52
Medical degree from non‐US/CA medical school	1.06 (0.82–1.38)	0.64	1.06 (0.82–1.38)	0.64
Missing	1.62 (0.58–4.55)	0.34	1.68 (0.60–4.70)	0.33
Board certification				
Yes	1.15 (0.81–1.63)	0.43	1.18 (0.83–1.66)	0.36
No	1 [Reference]	–	1 [Reference]	–
Academic affiliation				
Yes	1.35 (1.10–1.66)	0.004	1.36 (1.11–1.66)	0.003
No	1 [Reference]	–	1 [Reference]	–
*CME activities* g				
Professional meetings				
Participated	1.31 (1.07–1.60)	0.009		
Did not participate	1 [Reference]	–		
Journal‐based CME				
Participated	1.29 (1.07–1.56)	0.009		
Did not participate	1 [Reference]	–		
Online specialty‐based courses				
Participated	0.95 (0.78–1.15)	0.57		
Did not participate	1 [Reference]	–		
Local conferences				
Participated	1.01 (0.83–1.23)	0.90		
Did not participate	1 [Reference]	–		
In‐person specialty‐based courses				
Participated	1.20 (0.97–1.48)	0.09		
Did not participate	1 [Reference]	–		
Learning from teaching others				
Participated	1.00 (0.79–1.26)	0.99		
Did not participate	1 [Reference]	–		
Performance improvement				
Participated	0.97 (0.76–1.25)	0.83		
Did not participate	1 [Reference]	–		
Manuscript review				
Participated	1.49 (1.00–2.22)	0.05		
Did not participate	1 [Reference]	–		
Committee learning				
Participated	1.03 (0.74–1.42)	0.87		
Did not participate	1 [Reference]	–		
Test‐item writing				
Participated	1.01 (0.71–1.42)	0.97		
Did not participate	1 [Reference]	–		
Other CME activity				
Participated	0.82 (0.55–1.22)	0.32		
Did not participate	1 [Reference]	–		
Extent of CME‐activity participation^h^			1.10 (1.04–1.17)	0.001

*Note*: Logistic regression results of physicians who were satisfied versus not satisfied/neutral with their ability to stay current on medical information and practice. Model 1 includes demographic characteristics, professional attributes, and each of the 11 CME activities in which physicians participated in the past year. Model 2 includes demographic characteristics, professional attributes, and the extent of CME activities participated in the past year.

Abbreviations: AOR, adjusted odds ratio; CA, Canada; CI, confidence interval; CME, continuing medical education; URM, underrepresented in medicine; US, United States.

^a^ An analytical weight was applied to all analyses to ensure representativeness of all practicing physicians in the United States for specialty, gender, and age. Unweighted *N* = 5930.

^b^ Model 1 includes demographic characteristics, professional attributes, and each of 11 CME activities in which physicians participated in the past year. Archer–Lemeshow goodness‐of‐fit test: *p* = 0.926 suggests there is no evidence of lack of model fit. Regression diagnostics for multicollinearity among explanatory variables showed that all variance inflation factors were substantially below the threshold (values less than 10) to indicate that collinearity was a concern.

^c^ Each association was tested using *χ* ^2^ test of association and considered statistically significant when the *p* value was less than 0.05.

^d^ Model 2 includes demographic characteristics, professional attributes, and the extent of CME activities participated in the past year. Archer–Lemeshow goodness‐of‐fit test: *p* = 0.270 suggests there is no evidence of lack of model fit. Regression diagnostics for multicollinearity among explanatory variables showed that all variance inflation factors were substantially below the threshold (values less than 10) to indicate that collinearity was a concern.

^e^ “URM” includes Hispanic, Latino, or of Spanish origin; American Indian or Alaskan Native; Black or African American; or Native Hawaiian or other Pacific Islander alone or in combination with any other race/ethnicity. “Other” includes Other alone; Asian and White; Asian and Other; White and Other; or Asian, White, and Other in combination.

^f^ Top three specialties within “Other” include Psychiatry (14%, 252/1748), Anesthesiology (14%, 244/1748), and Emergency Medicine (13%, 219/1748).

^g^ Associations between participation in each CME activity and satisfaction were assessed in Model 1 only.

^h^ Since respondents were able to select multiple CME activities participated in the past year, we identified the total number of different activities in which respondents participated (mean = 3.35, SD = 1.73) and separately assessed the association with physician satisfaction in Model 2.

Findings for CME activities in each model were as follows. In Model 1, physicians who participated (vs. did not participate) in each of “professional meetings” (AOR, 1.31; 95% CI, 1.07–1.60) and “journal‐based CME” (AOR, 1.29; 95% CI, 1.07–1.56) were more likely to be satisfied with their ability to stay current. Satisfaction was not independently associated with participation in each of “online specialty‐based courses” (AOR, 0.95; 95% CI, 0.78–1.15), “local conferences” (AOR, 1.02; 95% CI, 0.83–1.23), “in‐person specialty‐based courses” (AOR, 1.20; 95% CI, 0.97–1.48), “learning from teaching others” (AOR, 1.00; 95% CI, 0.79–1.26), “performance improvement” (AOR, 0.97; 95% CI, 0.76–1.25), “manuscript review” (AOR, 1.49; 95% CI, 1.00–2.22), “committee learning” (AOR, 1.03; 95% CI, 0.74–1.42), “test‐item writing” (AOR, 1.01; 95% CI, 0.71–1.42), and “other” CME activity (AOR, 0.82; 95% CI, 0.55–1.22). In Model 2, satisfaction with ability to stay current increased by 10% for each additional CME‐activity type participated (AOR, 1.10; 95% CI, 1.04–1.17).

## 4 DISCUSSION

We explored physician satisfaction with the ability to stay current on medical information and practice, and the nature of participation in CME among a large sample of practicing physicians in the United States. We also analyzed independent relationships between satisfaction with the ability to stay current and each of several demographic variables (gender, age, and race/ethnicity) and each of several professional attributes (specialty category, medical degree type, board certification, and affiliation with an academic health system). Overall, practicing physicians in the United States were quite satisfied with their ability to stay current on medical information and practice at the time of this survey. We observed that physicians who participated in professional meetings and those who participated in journal‐based CME (reading one or more from a peer‐reviewed professional journal) were significantly more likely to be satisfied with their ability to stay current than those who were not engaged in each of these activities. These observations may be relevant to organizations and institutions that design and implement CME activities in the current context of COVID‐19 pandemic‐related in‐person activity limitations, especially as health professionals previously reported a preference for in‐person CME/CPD offerings.^5^, ^12^ Recent studies that have investigated physicians' perceptions of virtual CME alternatives prompted by the pandemic have also identified that participants prefer hybrid (face‐to‐face and online) formats and that virtual offerings should not replace but complement traditional in‐person offerings.^3^, ^13^, ^14^, ^15^ These perspectives, along with our observations, may help inform future planning of CME offerings to better support physicians' learning preferences in the context of COVID‐related impacts on the feasibility of offering particular types of CME activities.

Our observations regarding age are consistent with the thesis that mid‐career physicians (40–49 years old) likely experience challenges in work–life balance as they seek to raise families and advance professionally. In another study that surveyed US physicians to identify professional development priorities, practices, beliefs, and needs, longer practicing (>30 years since training) physicians had reported time as less of a barrier to professional development compared to physicians with less than 20 years since training.^6^ This mid‐career age group in particular could be a target for a range of highly flexible, on‐demand approaches to CME.

We examined physician race/ethnicity in association with our outcome of interest as recognition of racial/ethnic disparities in physician workforce training outcomes increases, particularly for URM physicians compared to white physicians. In our multivariable models, physicians who self‐identified as URM or “other” race/ethnicity (who comprised 4% of our study sample) were less likely to be satisfied with their ability to stay current compared to physicians who self‐identified as white. As consideration of physician race/ethnicity has been largely absent from CME research, the differences we observed in self‐reported satisfaction between self‐identified URM and white physicians can inform new lines of inquiry that are needed to better understand the causes and potential methods for addressing the disparity.

We identified two professional attributes independently associated with our outcome: specialty category and academic affiliation. Our specialty findings may be of interest to organizations and professional development programs focused on particular specialties. Our results suggest that approaches taken for those focused on surgeons appear to be particularly well‐received by participating physicians. Finally, we observed that being affiliated with an Academic Health Center or teaching hospital increased the likelihood of satisfaction with the ability to stay current. Thus, it is worth considering if there are CME benefits of academic affiliations that can be extended to physicians without such affiliations. As authors Rayburn, Regnier, and McMahon shared,^16^ there are clear and growing opportunities to leverage the trusted status of medical schools as *value* centers for continuous learning and improvement. The conversion of traditional face‐to‐face educational activities (e.g., “Grand Rounds”) to virtual events at academic institutions may offer opportunities to broadly expand physician access to such offerings^17^ that may be particularly well‐suited in the COVID‐19 environment.

Our study has numerous limitations. All data included in our study were self‐report only, which does not equate to actual performance. Although we conducted weighted analyses to ensure the generalizability of the results to all physicians nationally in the United States, there may have been selection bias among physicians who chose to participate in the survey regarding their participation in CME activities and reluctancy to report not being able to stay current on medical information and practice. Additionally, these results may not necessarily generalize to continuing educational/professional development in other health professions and to physicians outside the United States. Another limitation is the survey's measure of participation in CME activities in the form of a checklist (participation vs. nonparticipation), which does not reflect the intensity or time spent in each activity as different activities may require different levels of engagement. Furthermore, the extent to which there may be a relationship between physician satisfaction with their ability to stay current on medical information and practice and the quality of care delivered by the physician is unknown. Finally, it is important to note that the survey was administered to physicians in the pre‐COVID‐19 era; as with virtually every other aspect of physicians' professional lives, the accessibility and availability of many CME/CPD activities has been substantially altered. In this context, our study results serve as “baseline” data regarding physician perspectives pre‐COVID that can inform adaptations and modifications of CME/CPD in the current environment. We also observed significant differences in our outcome in association with race/ethnicity and age, suggesting that inclusion of these demographic characteristics in studies of other health professional groups may be similarly informative.

## 5 CONCLUSIONS

Overall these data, collected shortly before the onset of the COVID‐19 pandemic, identify that physicians were quite satisfied with their ability to stay current on medical information and practice. Independent associations that we observed for each age, race/ethnicity, specialty, and academic affiliation status, may inform the design and implementation of targeted CME/CPD activities. Our observations regarding various CME activities associated with satisfaction with the ability to stay current may be particularly relevant to CME/CPD providers and educators in future planning and strategies for CME/CPD offerings in the current context of COVID‐19 pandemic‐related restrictions and limitations of face‐to‐face activities. Further research is warranted to inform targeted interventions that can address the observed differences in satisfaction and to determine if there is a relationship between physicians' satisfaction with their ability to stay current on medical information, their actual practice, and the quality of care delivered.

## AUTHOR CONTRIBUTIONS

**Amy Jayas**: Conceptualization; data curation; formal analysis; investigation; methodology; project administration; software; visualization; writing—original draft; writing—review & editing. **Dorothy A. Andriole**: Conceptualization; investigation; methodology; project administration; supervision; writing—original draft; writing—review & editing. **Douglas Grbic**: Conceptualization; data curation; formal analysis; investigation; methodology; software; writing—original draft; writing—review & editing. **Xiaochu Hu**: Conceptualization; data curation; investigation; methodology; writing—review & editing. **Michael Dill**: Conceptualization; investigation; writing—review & editing. **Lisa D. Howley**: Conceptualization; investigation; methodology; supervision; writing—original draft; writing—review & editing.

## CONFLICT OF INTEREST STATEMENT

The authors declare no conflict of interest.

## TRANSPARENCY STATEMENT

Amy Jayas affirms that this manuscript is an honest, accurate, and transparent account of the study being reported; that no important aspects of the study have been omitted; and that any discrepancies from the study as planned (and, if relevant, registered) have been explained. All authors have read and approved the final version of the manuscript. Amy Jayas had full access to all of the data in this study and takes complete responsibility for the integrity of the data and the accuracy of the data analysis.

## Data Availability

Due to the nature of this research, participants of this study did not agree for their data to be shared publicly, so supporting data are not available.
